# Altered glucose metabolism rather than naive type 2 diabetes mellitus (T2DM) is related to vitamin D status in severe obesity

**DOI:** 10.1186/1475-2840-13-57

**Published:** 2014-03-11

**Authors:** Mattia Bellan, Gabriele Guzzaloni, Maura Rinaldi, Elena Merlotti, Carlotta Ferrari, Antonella Tagliaferri, Mario Pirisi, Gianluca Aimaretti, Massimo Scacchi, Paolo Marzullo

**Affiliations:** 1 Department of Translational Medicine, Division of Internal Medicine, Università del Piemonte Orientale “A. Avogadro”, Novara, Italy; 2 Division of General Medicine, Ospedale S. Giuseppe, I.R.C.C.S. Istituto Auxologico Italiano, Verbania, Italy; 3 Department of Translational Medicine, Università del Piemonte Orientale “A. Avogadro”, Novara, Italy; 4 Department of Clinical and Community Sciences, University of Milan, Milan, Italy

## Abstract

**Context:**

The last decades have provided insights into vitamin D physiology linked to glucose homeostasis. Uncertainties remain in obesity due to its intrinsic effects on vitamin D and glucose tolerance.

**Objectives:**

To assess the relationship between vitamin D and glucose abnormalities in severely obese individuals previously unknown to suffer from abnormal glucose metabolism.

**Setting:**

Tertiary care centre.

**Patients:**

524 obese patients (50.3 ± 14.9 yrs; BMI, 47.7 ± 7.3 kg/m^2^) screened by OGTT, HbA_1c_ and the lipid profile. Vitamin D status was assessed by 25(OH)D_3_, PTH and electrolyte levels. 25(OH)D_3_ deficiency/insufficiency were set at 20 and 30 ng/ml, respectively. All comparative and regression analyses were controlled for age, BMI and gender.

**Results:**

The prevalence of vitamin D deficiency/insufficiency and secondary hyperparathyroidism were 95% and 50.8%, respectively. Normal glucose tolerance (NGT), impaired fasting glucose (IFG) or impaired glucose tolerance (IGT), and type 2 diabetes mellitus (T2DM) were found in 37.8%, 40.5% and 21.7% of cases, respectively. Large variations in metabolic parameters were seen across categories of vitamin D status, but the only significant differences were found for C-peptide, tryglicerides, LDL- and HDL-cholesterol levels (p < 0.05 for all). The prevalence of vitamin D deficiency was documented to be slightly but significantly more frequent in glucose-intolerant patients (IFG + IGT + T2DM) compared to the -normotolerant counterpart (87% vs. 80%, p < 0.05). In partial correlation analyses, there was no association between vitamin D levels and glucose-related markers but for HbA_1c_ (r = −0.091, p < 0.05), and both basal and OGTT-stimulated insulin levels (r = 0.097 and r = 0.099; p < 0.05 for all). Vitamin D levels were also correlated to HDL-cholesterol (r = 0.13, p = 0.002). Multivariate regression analysis inclusive of vitamin D, age, BMI, gender and fat mass as independent variables, showed that vitamin D was capable of predicting HbA_1c_ levels (β = −0.101, p < 0.05).

**Conclusions:**

Given the inherent effect of obesity on vitamin D and glucose homeostasis, current data suggest a potential independent role for vitamin D in the regulation of glucose metabolism in a setting of obese patients previously unknown to harbour glucose metabolism abnormalities.

## Introduction

Vitamin D is a secosteroid hormone, with an established role in bone homeostasis. Its di-hydroxylated active metabolite [1], 1,25(OH)_2_D_3_, binds the vitamin D nuclear receptor (VDR) and translocates to the nucleus to regulate gene expression [2]. Being the VDR located in several cells and tissues, a number of new hypothetical functions have been postulated to expand vitamin D role beyond its ability to regulate calcium homeostasis. As such, VDR has been found to affect 229 human genes [3], and in vivo and in vitro studies have increasingly linked vitamin D homeostasis to cardiovascular, autoimmune, tumoral, pulmonary and neurological diseases [4-7].

Assessment of vitamin D status relies on plasma measurement of its circulating metabolite, 25-hydroxyvitamin D (25(OH)D_3_), which is more stable, has a longer half-life and reflects vitamin D storage more accurately than the active form [8]. Although no definitive consensus currently exists on the lowest 25(OH)D_3_ levels of normalcy, the 25(OH)D_3_ threshold of 30 ng/ml (75 nmol/l) is deemed as adequate for fracture prevention in the general older population [9-11], while the 20 ng/ml cutoff limit has been alternatively suggested to differentiate populations at true risk for the effects of vitamin D deficiency [12]. Cohort studies showed that vitamin D inadequacy occurs in approximately 36% of otherwise healthy young adults and up to 57% of inpatients, with even higher rates applying to European populations [13,14].

Causal factors of vitamin D deficiency include aging [15], longer life expectancy [16], lifestyle habits [17], and metabolic disorders [18]. An increased proportion of body fat as well as obesity have been documented to decrease bioavailability of cholecalciferol [19-21], due to its preferential accumulation in the adipose tissue [22]. Especially, a deranged vitamin D status may reflect an increased risk of type 2 diabetes mellitus (T2DM) in the general population [23]. While it is not completely clarified how vitamin D acts on glucose metabolism, postulated mechanisms include direct effects on insulin synthesis and release mediated by the VDR [24], and negative effects on insulin sensitivity elicited by secondary elevation of PTH levels [25]. As vitamin D upregulates lipoprotein lipase (LPL), this latter has been suggested to act as a potential link between vitamin D and glucose metabolism [26]. Nevertheless, the potential effects of vitamin D status on insulin sensitivity are debated, as the correlation between vitamin D and response to insulin has been found direct by some studies [27] and modestly significant by others [28]. Recently, Muscogiuri and colleagues found that the correlation between low vitamin D levels and insulin resistance could be influenced by obesity, which was demonstrated to be the only predictor of low vitamin D levels [29] by multivariate analysis.

In this study, we aimed at exploring the relationship between vitamin D status and glucose homeostasis following screening for T2DM in a population of obese patients previously undiagnosed with abnormalities of glucose metabolism. To this purpose, our aims were: to subject obese patients to screening of glucose tolerance and insulin resistance by the OGTT and HbA_1c_ levels, and evaluation of the lipid profile; to assess the prevalence of hypovitaminosis D and secondary hyperparathyroidism in severe obesity; to examine the relationship linking low vitamin D concentrations to glucose intolerance, T2DM and lipid markers after correction for key variables linked to these parameters; to identify a predictive role of vitamin D concentrations on glucose and lipid metabolism in this cohort.

## Material and methods

We performed a single centre study in severely obese patients referring to the Istituto Auxologico Italiano (Verbania, Italy) for work-up and rehabilitation of their obese status. Data presented herein are part of a larger screening study on the prevalence of previously undiagnosed glucose intolerance or incident T2DM in obesity (Type 2 diabetes mellitus Of New Diagnosis in Obesity, TONDO). More specifically, the TONDO study was designed to investigate the relationship between newly diagnosed glucose abnormalities and biochemical or functional markers of organ damage in subjects with uncomplicated and complicated obesity, with BMIs spanning from 35 to 70 kg/m^2^. Current data refer to an *ad interim* analysis on the relationship between vitamin D status and glucose metabolism. Approval from the Ethic Committee was obtained prior to the beginning of the study, and all patients signed the informed consent before enrollment into the study.

The current analysis included 524 consecutive caucasian subjects affected with grade II and III obesity (BMI ≥35 kg/m^2^), aged ≥ 18 years. Exclusion criteria were as follows: previously known T1DM and untreated or treated T2DM; hormone treatments including corticosteroids; any therapy capable of influencing calcium metabolism; previous or current vitamin D treatment; comorbidities affecting vitamin D metabolism, such as chronic kidney disease, liver cirrhosis, gastroenteropancreatic disturbances, autoimmune disorders, primary hyperparathyroidism.

Upon enrollment, data were resumed as follows: height, weight, BMI (kg/m^2^), waist and hip circumferences, waist-to-hip ratio (WHR), personal and family history, ongoing therapies, body composition. Routine laboratory data included levels of 25(OH)D_3_, PTH, calcium and phosphorus, and C-reactive protein. Glucose metabolism was assessed by fasting plasma glucose, insulin and C-peptide levels; oral glucose tolerance test (OGTT) for glucose and insulin levels; glycated haemoglobin (HbA_1c_). Lipid analysis included total-cholesterol (t-CHO), high density lipoprotein-cholesterol (HDL-CHO), low density lipoprotein -cholesterol (LDL-CHO) and triglycerides levels.

ADA recommendations [30] were used for the definition of glucose metabolism and T2DM, as follows: normal fasting plasma glucose (FPG) if <100 mg/dl (5.6 mmol/l); impaired FPG (IFG) if FPG was 100–125 mg/dl (6.9 mmol/l); impaired glucose tolerance (IGT) if 2-h post-OGTT plasma glucose was 140–199 mg/dl (7.8-11.0 mmol/l); T2DM if FPG was ≥126 mg/dl (≥7 mmol/l) on two days apart, or if 2-h post-OGTT plasma glucose was ≥200 mg/dl (≥11.1 mmol/l). HbA_1c_ values of 5.7 and 6.5% were considered as the threshold of normal glucose metabolism and T2DM, respectively. Insulin resistance was calculated by the homeostatic model of insulin resistance (HOMA-IR) as fasting insulin (μU/m) × [fasting PG (mmol/l)/22.5]. Vitamin D status was defined according to current guidelines [11] as: deficiency, 25(OH)D_3_ < 20 ng/ml; insufficiency, 25(OH)D_3_ 20–29 ng/ml; adequacy, 25(OH)D_3_ ≥ 30 ng/ml.

Waist was measured as halfway between the costal edge and the crista. Hip was measured as the greatest circumference around the nates.

Body composition was evaluated according to percentage of fat and fat-free mass. Fat mass (FM), fat-free mass (FFM), total body water, and extracellular water (ECW) were determined by bioelectrical impedance analysis (BIA 101/S; Akern, Florence, Italy). Analysis was performed using Bodygram software version 1.2 (Akern).

Laboratory data were obtained in a central laboratory. Blood glucose, t-CHO, HDL-CHO, LDL-CHO and triglycerides, and HbA_1c_ were measured by enzymatic methods (Roche Molecular Biochemicals, Mannheim, Germany). A two-site, solid-phase chemiluminescent immunometric assay or competitive immunoassay (Immulite 2000 Analyzer; DPC, Los Angeles, CA) was used for C-peptide with intra- and interassay coefficients of variation (CVs) of 1.9-3.3% and 3.8-5.5%, and PTH levels (CVs, 4.2-5.7% and 6.3-8.8%). Levels of 25(OH)D_3_ (CVs, 1.7-7.8 and 2.2-10.7%) and insulin (CVs, 0.8-1.5% and 2.4-4.9%) were measured using a Cobas Integra 800 Autoanalyzer (Roche Diagnostics, Indianapolis, IN). Ultrasensitive C-reactive protein was measured by (latex) HS Roche kit using Cobas Integra 800 (Roche Diagnostics).

## Statistical analysis

Statistical analyses were performed using IBM SPSS (version 18, Somers, NY, USA). The statistical significance standard was set at 5%. Data normality was tested using the Shapiro Wilk test. If data points were not normally distributed, statistical analysis was attempted on the natural logarithm of the values to improve the symmetry and homoscedasticity of the distribution. Log-transformed values of 25(OH)D_3_, PTH, glucose, insulin, c-peptide, C-reactive protein, HbA_1c_, calcium and phosphorus were therefore used for comparative, univariate and multivariate analyses. For homogeneity of presentation, both normally distributed and non-normally distributed data are presented as medians with interquartile ranges (IQR). Differences in categorical variables were analyzed by *χ*^2^ test, and two-tailed, unpaired T- test or analysis of covariance were used for continuous variables depending on their distribution. Regression analyses and analysis of variance for multiple dependent variables by one or more factor variables or covariates were calculated using the general linear model multivariate procedure, comprising of the effects of covariate interactions with the variables of interest. To determine the impact of vitamin D status on metabolic variables, different models were attempted and the following covariates of interest, as well as their interaction, were used if not mutually excluded by the collinearity test: age, gender, BMI, percent fat body mass, fat body mass in weight, lean body mass in weight, and WHR. Multivariate linear and logistic regression analysis were used to estimate the coefficients of the linear equation, involving one or more independent variables, that best predict the value of the dependent variable.

## Results

Data from 524 severely obese patients (303 females; 221 males), consecutively recruited according to the inclusion criteria, constituted our analytical sample, as detailed in Table 1. After subgrouping by gender, there were expected differences in anthropometric and metabolic parameters, whilst 25(OH)D_3_ and PTH levels were not dissimilar between men and women. In the population as a whole, screening for T2DM by the OGTT and HbA_1c_ levels revealed normal glucose metabolism in 37.8%, while IFG / IGT were present in 40.5%; de novo T2DM was diagnosed in 21.7% of subjects (Table 2). Analysis of 25(OH)D_3_ levels showed no seasonal variations, and 25(OH)D_3_ concentrations were in the range of deficiency in 84.7% (N = 444; F/M, 57.4/42.6%), insufficiency in 10.3% (N = 54; F/M, 58.2/41.8%) and normalcy in 5% of patients (N = 26; F/M, 64.0/46.0%). The distribution of vitamin D status was similar between genders. Although the prevalence of glucose alterations differed markedly across categories of vitamin D status (Table 3), the only difference achieving statistical significance was documented for C-peptide levels. Of note, analysis of the lipid panel underscored significant differences in HDL-CHO, LDL-CHO and triglycerides levels in function of vitamin D status (Table 3). When vitamin D status was analyzed in function of glucose metabolism we could only observe nonsignificant differences across groups after controlling for age, BMI and gender (Table 4). Oppositely, when the propensity toward vitamin D deficiency was analyzed in function of glucose metabolism controlled for age, BMI and gender, we found that the prevalence of vitamin D deficiency was slightly but significantly greater (87% vs. 80%; OR: 1.75, 95% CI: 1.03-2.99; p = 0.04) in patients with an abnormal glucose profile (IFG + IGT + T2DM) compared to those with normal tolerance.

**Table 1:** Anthropometric and analytical variables obtained in the study population

**Parameters**	**Whole population**	**Males**	**Females**	**P**
	**(N = 524)**	**(N = 221)**	**(N = 303)**	
**Age (years)**	52.0 [40.0-62.0]	47.0 [39.0-59.0]	54.0 [41.0-64.0]	0.001
**BMI (kg/m**^**2**^**)**	46.6 [43.1-50.9]	46.8 [42.6-51.2]	46.3 [43.3-50.8]	0.001
**WHR**	0.94 [0.89-1.0]	1.02 [0.98-1.08]	0.90 [0.85-0.93]	0.0001
**Fat mass (%)**	50.0 [44.1-54.3]	42.2 [38.3-46.0]	53.4 [50.3-56.2]	0.0001
**C-reactive protein (mg/dl)**	0.6 [0.4-1.1]	0.5 [0.3-1.9]	0.7 [0.4-1.2]	0.0001
**FPG (mg/dl)**	96.0 [90.0-107.0]	98.0 [90.0-110.5]	95.0 [89.0-105.0]	0.0002
**Post-OGTT glucose (mg/dl)**	143.0 [112.0-178.0]	157.0 [125.0-187.0]	135.0 [107.0-164.0]	0.0001
**Fasting insulin (μU/ml)**	13.1 [8.9-20.0]	15.5 [10.3-24.1]	12.3 [7.9-17.4]	0.001
**Post-OGTT insulin (μU/ml)**	89.0 [56.5-138.6]	102.3 [69.6-164.7]	77.4 [50.7-123.6]	0.0001
**Fasting C-peptide (μg/l)**	3.6 [2.8-4.6]	3.9 [3.2-5.0]	3.4 [2.7-4.3]	0.0001
**HbA**_**1c **_**(%)**	5.7 [5.5-6.0]	5.7 [5.5-6.1]	5.7 [5.5-6.0]	0.005
**HOMA-IR**	3.2 [2.0-4.9]	3.5 [2.3-5.9]	2.9 [1.9-4.2]	0.0002
**25(OH)D**_**3 **_**(ng/ml)**	10.3 [9.0-15.8]	10.8 [9.0-17.0]	10.0 [9.0-15.2]	0.98
**PTH (pg/ml)**	65.5 [48.2-90.4]	64.3 [48.0-91.8]	66.0 [48.5-89.5]	0.88
**Phosphorus (mg/dl)**	3.6 [3.3-4.0]	3.6 [3.2-4.0]	3.7 [3.3-4.1]	0.0005
**Calcium (mg/dl)**	9.1 [8.8-9.4]	9.1 [8.8-9.4]	9.1 [8.8-9.4]	0.93
**Triglycerides (mg/dl)**	131.0 [101.7-172.0]	142.0 [109.0-184.0]	126.0 [94.0-167.0]	0.0001
**Total cholesterol (mg/dl)**	218.0 [191.0-247.4]	188.0 [165.0-213.0]	193.0 [172.0-221.0]	0.11
**LDL cholesterol (mg/dl)**	144.0 [122.0-171.0]	120.0 [101.0-145.0]	123.0 [104.0-144.0]	0.78
**HDL cholesterol (mg/dl)**	50.0 [42.0-62.0]	37.0 [31.0-43.0]	45.0 [38.0-57.0]	0.0001
**Patients with NGT (%)**	37.8	26.2	46.2	0.0001
**Patients with IFG-IGT (%)**	40.5	45.2	37.0	0.05
**Patients with T2DM (%)**	21.7	28.5	16.8	0.001

Data are presented as median values and interquartile ranges. For comparative analysis between genders, significance is calculated by unpaired *T* test for age and BMI, and by analysis of covariance for all other variables with age and BMI as covariates. The *χ*^2^ test was used for comparative analyses between patients with NGT, IFG + IGT and T2DM. For abbreviations: BMI, Body Mass Index; WHR, Waist-to-Hip Ratio; FPG, Fasting Plasma Glucose; HbA_1c_, Glycated Haemoglobin; HOMA-IR, Homeostatic Model of Insulin Resistance; 25(OH)D_3_, 25-hydroxy-vitamin D; PTH, Parathyroid Hormone; NGT, Normal Glucose Tolerance; IFG, Impaired Fasting Glucose; IGT, Impaired Glucose Tolerance; T2DM, Type 2 Diabetes Mellitus.

**Table 2:** Patients distribution according to vitamin D status and glucose homeostasis

	**25(OH)D**_**3 **_**deficiency**	**25(OH)D**_**3 **_**insufficiency**	**25(OH)D**_**3 **_**adequacy**	**Total**
**NGT**	160 (30.5%)	28 (51.8%)	10 (38.5%)	198 (37.8%)
**IFG/IGT**	184 (35.1%)	17 (31.5%)	11 (42.3%)	212 (40.5%)
**T2DM**	100 (19.1%)	9 (16.7%)	5 (19.2%)	114 (21.7%)
**Total**	444 (84.7%)	54 (10.3%)	26 (5.0%)	524 (100%)

Data are presented as cumulative values with percentages in parentheses. For abbreviations: 25(OH)D_3_, 25-hydroxyvitamin D; PTH, Parathyroid Hormone; NGT, Normal Glucose Tolerance; IFG, Impaired Fasting Glucose; IGT, Impaired Glucose Tolerance; T2DM, Type 2 Diabetes Mellitus.

**Table 3:** Differences in continuous variables according to vitamin D status

	**25(OH)D**_**3 **_**deficiency**	**25(OH)D**_**3 **_**insufficiency**	**25(OH)D**_**3 **_**adequacy**	**p**
	**(N = 444)**	**(N = 55)**	**(N = 25)**	
**Age (years)**	53.0 [40.0-61.0]	52.5 [38.7-64.0]	52.0 [46.2-68.0]	0.78
**BMI (kg/m**^**2**^**)**	46.7 [43.3-51.3]	46.4 [41.2-49.0]	43.4 [40.8-47.1]	0.01
**WHR**	0.94 [0.89-1.02]	0.93 [0.88-1.01]	0.97 [0.90-1.03]	0.33
**Fat mass (%)**	49.6 [44.3-54.5]	50.0 [41.9-53.9]	51.0 [41.8-53.5]	0.59
**C-reactive protein (mg/dl)**	0.6 [0.4-1.1]	0.5 [0.3-1.0]	0.5 [0.4-1.0]	0.59
**FPG (mg/dl)**	96.0 [90.0-107.0]	93.0 [86.7-101.2]	97.0 [91.5-111.2]	0.06
**Post-OGTT glucose (mg/dl)**	144.0 [113.0-178.0]	125.0 [104.5-171.0]	136.0 [97.5-185.5]	0.29
**Fasting insulin (μU/ml)**	12.9 [8.6-19.8]	15.3 [10.0-20.9]	12.8 [8.6-25.4]	0.08
**Post-OGTT insulin (μU/ml)**	89.9 [55.1-138.8]	81.0 [57.4-135.6]	78.2 [52.9-135.0]	0.91
**Fasting C-peptide (μg/l)**	3.6 [2.8-4.6]	3.2 [2.5-4.9]	4.0 [3.0-5.9]	0.04
**HbA**_**1c **_**(%)**	5.8 [5.5-6.1]	5.6 [5.4-5.9]	5.7 [5.4-6.0]	0.10
**HOMA-IR**	3.1 [2.0-5.0]	3.6 [2.2-4.8]	3.3 [1.9-5.3]	0.14
**PTH (pg/ml)**	67.7 [50.0-93.2]	52.4 [39.6-70.8]	60.2 [34.2-74.2]	0.002
**Phosphorus (mg/dl)**	3.6 [3.3-4.0]	3.6 [3.1-3.8]	3.7 [3.5-4.1]	0.44
**Calcium (mg/dl)**	9.1 [8.8-9.4]	9.2 [8.9-9.5]	9.3 [9.0-9.5]	0.20
**Triglycerides (mg/dl)**	133.0 [102.0-174.2]	122.5 [91.0-155.0]	130.0 [101.0-168.0]	0.02
**Total cholesterol (mg/dl)**	192.0 [169.0-218.2]	182.0 [165.5-205.7]	200.0 [167.7-233.5]	0.09
**LDL cholesterol (mg/dl)**	122.0 [102.0-144.0]	110.5 [91.2-139.7]	126.0 [104.0-151.2]	0.04
**HDL cholesterol (mg/dl)**	41.5 [34.0-49.2]	46.5 [38.0-56.2]	41.5 [33.2-55.7]	0.04

Data are presented as median values and interquartile ranges. For comparative analysis, significance is calculated by ANOVA for age and BMI, and by analysis of covariance for all other variables using age, BMI and gender as covariates. Categories of Vitamin D status are detailed in the Patients and Methods section. For abbreviations: BMI, Body Mass Index; WHR, Waist-to-Hip Ratio; FPG, Fasting Plasma Glucose; HbA_1c_, Glycated Haemoglobin; HOMA-IR, Homeostatic Model of Insulin Resistance; 25(OH)D_3_, 25-hydroxy-vitamin D; PTH, Parathyroid Hormone; NGT, Normal Glucose Tolerance; IFG, Impaired Fasting Glucose; IGT, Impaired Glucose Tolerance; T2DM, Type 2 Diabetes Mellitus.

**Table 4:** Difference in continuous variables between patients aggregated by glucose homeostasis

	**NPG + NGT**	**IFG + IGT + T2DM**	**P**
	**(N = 198)**	**(N = 326)**	
**Age (years)**	45.0 [33.0- 58.2]	55.0 [44.0-62.7]	0.001
**BMI (kg/m**^**2**^**)**	46.3 [43.3-50.0]	46.7 [43.0-51.1]	0.91
**WHR**	0.91 [0.86-1.00]	0.97 [0.90-1.04]	0.13
**Fat mass (%)**	51.0 [47.9-54.8]	48.3 [42.2-54.0]	0.18
**C-reactive protein (mg/dl)**	0.6 [0.4-1.1]	0.6 [0.3-1.1]	0.56
**25(OH)D**_**3 **_**(ng/ml)**	11.0 [9.0-17.6]	9.9 [9.0-15.0]	0.23
**PTH (pg/ml)**	62.6 [45.9-85.0]	67.7 [50.2-96.1]	0.22
**Phosphorus (mg/dl)**	3.7 [3.4-4.0]	3.6 [3.2-4.0]	0.95
**Calcium (mg/dl)**	9.1 [8.8-9.4]	9.1 [8.8-9.4]	0.80

Data are presented as median values and interquartile ranges. For comparative analysis, significance is calculated by ANOVA for age and BMI, and by analysis of covariance for all other variables using age, BMI and gender as covariates. For abbreviations: NPG, Normal Plasma Glucose; NGT, Normal Glucose Tolerance; IFG, Impaired Fasting Glucose; IGT, Impaired Glucose Tolerance; T2DM, Type 2 Diabetes Mellitus; BMI, Body Mass Index; WHR, Waist-to-Hip Ratio; 25(OH)D_3_, 25-hydroxy-vitamin D; PTH, Parathyroid Hormone; CRP, C-reactive protein.

Analysis of PTH levels revealed secondary hyperparathyroidism (PTH ≥65 pg/ml) in 50.8% of obese patients as a whole, and 54.7% of those with vitamin D deficiency/insufficiency. Having vitamin D levels in the range of deficiency (<20 ng/ml) significantly increased the risk of secondary hyperparathyroidism (O.R., 2.38; 95% CI, 1.38-4.1; p < 0.01). PTH concentrations tended to increase proportionately to abnormalities of glucose metabolism (Table 4).

**Figure 1:**
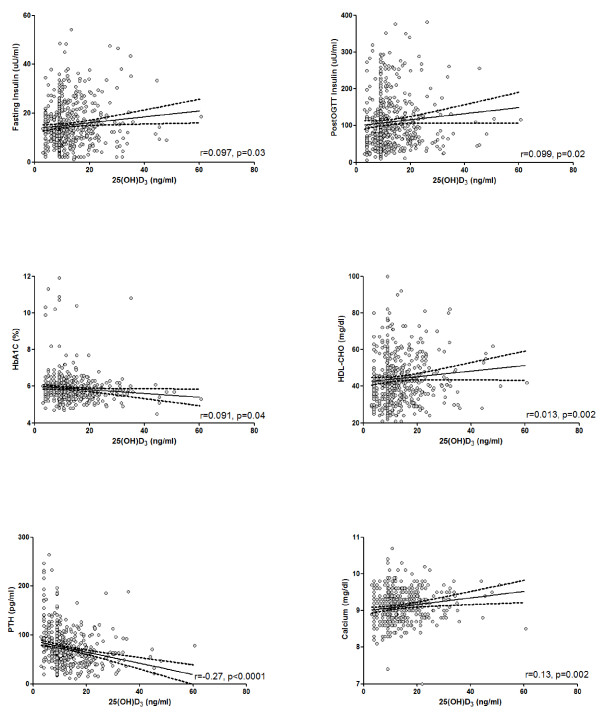
**Univariate regression analysis between metabolic variables of interest and vitamin D levels.** Individual data represent continuous variables. Regression coefficients were calculated by the general linear model on individual log-transformed values after controlling for age, BMI and gender. For abbreviations: 25(OH)D_3_, 25-hydroxyvitamin D; HbA_1c_, Glycated Haemoglobin; PTH, Parathyroid Hormone; OGTT, Oral Glucose Tolerance Test; HDL-CHO, HDL Cholesterol.

Correlation analysis was carried out after adjusting for age, BMI, and gender (Figure 1). We found no correlation between 25(OH)D_3_ and either baseline or post-OGTT glucose levels, while HbA_1c_ levels increased progressively with declining 25(OH)D_3_ levels (r = −0.091, p = 0.04). Of note, 25(OH)D_3_ concentrations were related to insulin levels by direct association, both in fasting conditions (r = 0.097, p = 0.03) and after OGTT (r = 0.099, p = 0.02), with near-significant associations to HOMA-IR (r = 0.085, p = 0.053) and c-peptide levels (r = 0.084, p = 0.06). A positive association was seen between 25(OH)D_3_ and HDL-CHO levels (r = 0.13, p = 0.002). In addition to the previous, 25(OH)D_3_ concentrations were markedly correlated to PTH (r = −0.27, p < 0.0001) and calcium levels (r = 0.13, p = 0.002).

Multiple logistic regression analysis was conducted on patients grouped by their glucose homeostasis as tolerant (NPG + NGT) and intolerant (IFG + IGT + T2DM). A correct vitamin D status was found to reduce the risk of glucose alterations (OR: 0.59; 95% CI: 0.36-1.00; p < 0.05), although aging (OR: 1.04; 95% CI: 1.02-1.05; p < 0.0001) and female gender (OR: 0.33; 95% CI: 0.22-0.50; p < 0.0001) played a more dominant role on such event. However, the effect of vitamin D was lost upon inclusion of BMI in the regression equation.

In stepwise multivariate regression analysis, vitamin D levels showed the ability to predict HbA_1c_ levels (β = −0.101, p = 0.026) after age (β =0.213, p = 0.001) and WHR (β = 0.207, p = 0.001). Neither BMI nor gender entered the regression equation. Vitamin D did not elicit any predictive effect on the remaining indices of glucose metabolism. When PTH replaced vitamin D levels as a continuous variable in analysis of covariance and in linear or logistic regression equations, we failed to observe any significant effect of PTH levels on glucose and lipid metabolism after controlling for key covariates used throughout the data analysis procedures.

## Discussion

This study, undertaken to assess if a causal relationship links glucose homeostasis and naive T2DM to vitamin D status in obesity, documented a negative effect of vitamin D deficiency on glycated haemoglobin when the effect of age, BMI, and gender was accounted for. Our analysis did not capture a direct association between diabetes mellitus and vitamin D, possibly due to the overwhelming effects of obesity on both conditions, however patients with impaired glucose tolerance clustered more frequently with an altered vitamin D status, after controlling for potential confounding factors. The potential relationship linking vitamin D to metabolic homeostasis in obesity was substantiated by the observed association between vitamin D and insulin secretion, as well as HDL cholesterol levels.

Evidence collected until now shows that the relationship between T2DM and hypovitaminosis D is debatable, as the bulk of data is based on observational or epidemiological studies, which are useful for generating hypotheses but not for proving causality [31]. In previous studies, lower vitamin D concentrations were independently associated with obesity, metabolic syndrome and IFG in a teenager population, with the risk of IFG being doubled in patients at the lowest compared to those at the highest quartile of vitamin D [32]. Similarly, waist circumference, triglycerides, fasting glucose and insulin sensitivity impairment were predicted by low vitamin D levels in an adult cohort of Australian patients [33]. Data from adults showed that hypovitaminosis D is correlated with the development of IGT [34], and an inverse correlation has been described between vitamin D concentrations and the risk of developing T2DM in a 22-year follow-up Finnish cohort study [35]; in obstructive sleep apnea syndrome-patients, vitamin D concentrations have also been inversely related to the risk of diabetes and metabolic syndrome [36]. Likewise, a higher prevalence of hypovitaminosis D was reported in diabetic patients compared to healthy controls in a population of middle aged Caucasian men and women and in South Asian UK residents [37,38]. Prospective studies prompted an inverse association between 25(OH)D_3_ levels and future glycemia and insulin resistance [39]. Finally, vitamin D seems to play a protective role on macrovascular damage in murine models of diabetes, slowing down one of the main diabetes related complications [40].

To the best of our knowledge, this is the first large study investigating such relationship in a homogeneous subset of severely obese patients, subjected to screening for T2DM by different biochemical approaches. One main finding consisted in the observation that neither 25(OH)D_3_ concentrations were lower nor hypovitaminosis D was significantly more frequent in prediabetic and diabetic obese patients compared to those with normoglycaemia, likely due to the blunting effect of obesity on circulating vitamin D levels. At variance with previous reports [32,34], we were unable to disclose negative effects of low 25(OH)D_3_ concentrations on plasma glucose levels both in fasting conditions and after the oral glucose challenge after correction for multiple variables known to affect glucose metabolism. Even so, we confirm the inverse correlation between 25(OH)D_3_ and glycated haemoglobin, an established marker of glucose homeostasis [30,41,42]. It would be therefore important to expand to obesity results of prospective cohort studies describing an inverse association between vitamin D levels and the odds of transitioning from normoglycaemia to IFG, from normoglycaemia to T2DM and from IFG to T2DM [43], confirmed by a recent meta-analysis [44]. Another circumstance suggestive of this association involves the direct correlation found between 25(OH)D_3_ and insulin levels, both on fasting and after the OGTT. In previous studies on individuals at risk for T2DM [45], 25(OH)D_3_ concentrations were independently associated to insulin sensitivity and beta-cell function, and in subjects at risk or not for T2DM a positive correlation was found between vitamin D and early response of C-peptide and insulin levels to the oral glucose challenge [46]. Our findings may thus support the notion that vitamin D yields regulatory effects on insulin secretion in vitro and in vivo [47-49], while being in apparent contrast with previous inconclusive studies on poorly-controlled T2DM-patients with exhausted insulin secretion [50]. Because our patients harboured a glucose tolerance that spanned from normal to naive T2DM, and may have thus retained a superior beta-cell activity than patients with chronic T2DM, our results support the findings of Harris et al., who studied the effects of vitamin D supplementation in prediabetic obese patients and showed that the increase in 25(OH)D_3_ concentrations was associated with an increase of insulin secretion rate and C-peptide concentrations [51]. In a study by Guasch et al. [52], high 25(OH)D_3_ levels were significantly associated with HDL-cholesterol levels and diabetes/hyperglycemia, but this relationship was lost after adjustment for BMI. Also, vitamin D supplementation was shown to improve the metabolic profile of diabetic Saudi individuals undergoing different therapeutic regimens, with particular effectiveness on HDL-cholesterol levels [53]. In our analysis, the lipid profile clearly reflected vitamin D status, and high 25(OH)D_3_ levels were significantly associated with higher levels of HDL cholesterol, after adjustment for key confounding factors. This seems to confirm that vitamin D status is inversely related to atherogenic dyslipidemia [54,55], and indirectly suggests that vitamin D may be independently protective against the atherogenic profile in a population at high risk for cardiovascular disease.

Finally, current results substantiate the known high prevalence of vitamin D deficiency associated with obesity; the reason for this association has not been fully defined, and is commonly attributed to vitamin D accumulation in the adipose tissue [22,56-61]. In our experience, the cumulative prevalence of vitamin D deficiency and insufficiency was 95%. In our clinical practice, patients diagnosed with vitamin D deficiency receive cholecalciferol treatment (300.000 units p.o. for two consecutive days) and undergo regular follow-up. Also frequent was secondary hyperparathyroidism, affecting nearly 55% of subjects with vitamin D deficiency/insufficiency. These figures consolidate those found in similar studies [62,63]. Peculiarly, we observed an inverse relationship between 25(OH)D_3_ and PTH levels on one hand, and calcium concentrations on the other, both confirming the secondary origin of hyperparathyroidism. In multivariate analysis, the enhancing effect of hypovitaminosis D on PTH secretion was independent of common confounding factors herein associated. It is important to note that growing attention has recently focused on PTH as being a potentially closer factor associated to metabolic abnormalities than vitamin D levels. PTH plays a role in increasing the cardiovascular risk possibly via its effects on blood pressure, insulin resistance, hyperglycaemia and low HDL-CHO levels [64]. In a population-based cross-sectional study of US men and women, the odds ratio for metabolic syndrome increased with increasing PTH in older men only [64], while a survey in aging European population showed that a decreased risk for metabolic syndrome with increasing quintiles of 25(OH)D_3_ but not with PTH [65]. Neither of these studies was adjusted for BMI. In another study in obese subjects, PTH was paradigmatically associated to the metabolic syndrome via other biomarkers like vitamin D and magnesium, albeit this association was only significant in women [66]. Based on our analysis in severely obese patients, our results do not appear to confirm the predictive role of PTH on metabolic derangement when BMI, age and gender are accounted for. It would be valuable to corroborate this observation in a study encompassing a wider BMI range.

The cross-sectional design of our study represents its main limitation, since no cause-effect relationship could have been investigated. Further studies are required to better define the impact of vitamin D status on the development of glucose metabolism alterations. Furthermore we have no data on the effect of vitamin D supplementation on glycated haemoglobin. Recently, the correction of poor vitamin D status has been inversely associated with fasting insulin and HOMA-IR, in obese adolescents, but the effect on HbA_1c_ levels has not been investigated [67]. An interventional study should specifically address this issue. Finally, if, on one hand, the high homogeneity of our sample could be considered a strength, on the other hand it could be seen as a weakness, since the selection of severely obese patients could have masked the relationship between vitamin D status and glucose metabolism. Some of the strengths of this study should not be neglected, including the significant amount of clinical data collected in a large population of obese people, the status of previously undiagnosed glucose abnormalities, and the adjustment for common confounders such as age, BMI, and gender.

## Conclusions

While confirming the high prevalence of hypovitaminosis D in severe obesity, current data suggest a possible role for vitamin D in the regulation of glucose tolerance, insulin secretion and lipid metabolism, on which obesity plays *per se* a dominant effect. Indirectly, our findings support the importance of vitamin D in contributing to metabolic homeostasis in obesity.

## Competing interests

The authors have no potential competing interest to disclose.

## Authors’ contributions

MB contributed to data analysis and interpretation, and wrote the manuscript; GG and MR contributed to the study plan, patients recruitment and analysis, and data interpretation; EM, CF and AT contributed to patients recruitment and data collection; MP, GA and MS contributed to data interpretation and discussion; PM contributed to study plan and project management, patients recruitment and data analysis, manuscript writing. All authors read and approved the final manuscript.
